# A Ribosomal Perspective on Neuronal Local Protein Synthesis

**DOI:** 10.3389/fnmol.2022.823135

**Published:** 2022-02-23

**Authors:** Sudhriti Ghosh Dastidar, Deepak Nair

**Affiliations:** Centre for Neuroscience, Indian Institute of Science, Bengaluru, India

**Keywords:** neurons, synapse, local translation, ribosome remodeling, *in vivo* dynamics

## Abstract

Continued mRNA translation and protein production are critical for various neuronal functions. In addition to the precise sorting of proteins from cell soma to distant locations, protein synthesis allows a dynamic remodeling of the local proteome in a spatially variable manner. This spatial heterogeneity of protein synthesis is shaped by several factors such as injury, guidance cues, developmental cues, neuromodulators, and synaptic activity. In matured neurons, thousands of synapses are non-uniformly distributed throughout the dendritic arbor. At any given moment, the activity of individual synapses varies over a wide range, giving rise to the variability in protein synthesis. While past studies have primarily focused on the translation factors or the identity of translated mRNAs to explain the source of this variation, the role of ribosomes in this regard continues to remain unclear. Here, we discuss how several stochastic mechanisms modulate ribosomal functions, contributing to the variability in neuronal protein expression. Also, we point out several underexplored factors such as local ion concentration, availability of tRNA or ATP during translation, and molecular composition and organization of a compartment that can influence protein synthesis and its variability in neurons.

## Introduction

Careful decoding of mRNA messages is a fundamental yet complex challenge for any cell. Highly arborized morphology of neurons demands precise spatial and temporal control of mRNA translation in response to synaptic or network activation (Kapur et al., 2017; Rangaraju et al., 2017; Holt et al., 2019). The last few decades of research have pointed out that mRNA translation is regulated locally in neuronal dendrites, axons, dendritic spines, presynaptic boutons, or axonal growth cones (Biever et al., 2019; Fernandopulle et al., 2021). In matured neurons, the cumulative extent of dendritic translation exceeds that of cell soma, allowing spatial variability required for network integrity and homeostasis in the brain (Job and Eberwine, 2001; Hanus and Schuman, 2013; Spillane et al., 2013). Other contemporary studies have confirmed that variability in local protein synthesis is critical for neuronal development, maintenance, synaptic signaling, and plasticity (Holt et al., 2019; Fernandopulle et al., 2021). Studies also suggest that disruptions of activity-induced local translation have drastic consequences leading to cognitive deficits observed in several neurodevelopmental disorders and neurodegenerative diseases (Bear et al., 2004; Oddo, 2012; Deshpande et al., 2020; Ma, 2020).

Ribosomes are the central components of the translation machinery (Baßler and Hurt, 2019). For a long time, all cytosolic ribosomes were believed to act similarly, following a sequence of well-defined steps (Ramakrishnan, 2002; Wilson and Cate, 2012). However, in the past 5 years, an emerging body of evidence indicates the presence of significant heterogeneity in cellular ribosomes (Genuth and Barna, 2018; Gay et al., 2022). In addition, translation factors often shape ribosomal performance by controlling numerous aspects such as subunit loading on mRNAs, translational fidelity, ribosomal processivity, speed of translocation, probability of reloading, and more (Jackson et al., 2010; Kapur et al., 2017; Neelagandan et al., 2020). Within neurons, ribosomes are produced in the nucleolus and transported to the cytosol (Stoykova et al., 1985; Baßler and Hurt, 2019). A fraction of this population is then sorted to distant locations from the cell soma to participate in local translation (Tiedge and Brosius, 1996; Scarnati et al., 2018; Hafner et al., 2019; Koltun et al., 2020). Further, remodeling of existing ribosomes can occur in remote locations in a biogenesis-independent fashion (Sotelo-Silveira et al., 2006; Shigeoka et al., 2019; Mofatteh, 2020; Fernandopulle et al., 2021). Such remodeling events together with other parameters such as local ion concentrations, mRNA and tRNA availability, steady-state ATP content, and signaling pathways govern ribosomal activity and translation in neuronal compartments (Holt and Schuman, 2013; Rangaraju et al., 2017; Biever et al., 2019, 2020; Holt et al., 2019).

Here, we review the current understanding of ribosome biogenesis and heterogeneity within a neuron. We highlight the mechanisms and local parameters that influence stochasticity in ribosomal performance. Also, we discuss the importance of studying ribosomal dynamics to explain this translational variability required for neuronal functions.

## Regulation of Ribosome Biogenesis Within Neurons

**FIGURE 1:**
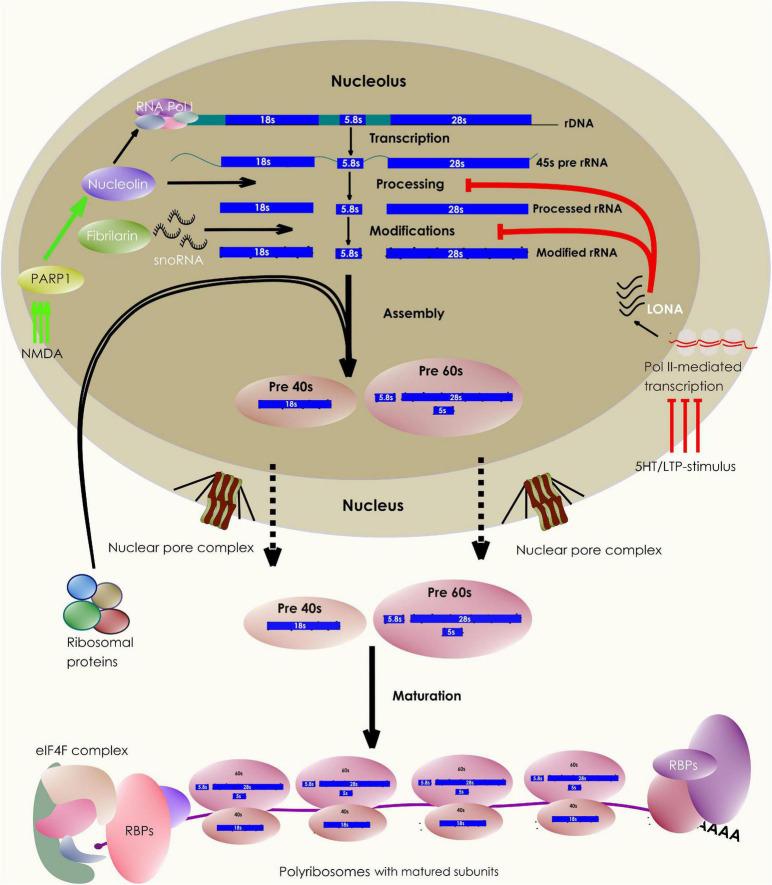
Neuronal control of ribosome biogenesis. The schematic depicts various stages of ribosome biogenesis within the nucleolar compartment. The rRNA synthesis, modification, and maturation takes place in the nucleolus following which, they associate with ribosomal proteins produced in the cytosol. The assembled subunits are then exported out of the nucleus to undergo the final steps of cytoplasmic maturation. A pool of these matured subunits engages in translating mRNAs, a process facilitated by multiple other protein factors. Interestingly, the nucleolar biogenesis process can be influenced by synaptic stimulation through a variety of synapse to nucleus (retrograde) signaling pathways.

“Ribosome Biogenesis” results in the production of functionally matured ribosomes that determines the protein synthetic ability of a living cell (Stoykova et al., 1985; Chau et al., 2018; Hetman and Slomnicki, 2019; Figure 1). The biogenesis process happens primarily in the nucleolus and is guided at least by 200 associating factors (AFs) and 80 different types of small nucleolar RNAs (snoRNA) (Baßler and Hurt, 2019).

Within the brain, three of the four rRNA species are synthesized together as a single polycistronic ∼45s rRNA precursor (pre rRNA) (Thomson et al., 2013; Baßler and Hurt, 2019). In general, the ribosomal DNA (rDNA) genes are present in multiple copies within the genome grouped into 7 different clusters, known as variants. Of these variants, 5 are expressed in the brain (Tseng et al., 2008; Allen et al., 2018). However, the rRNA content in individual brain cells can vary in a cell-type-specific manner. For example, studies have revealed that rat neurons possess fourfold higher pre rRNA content than oligodendrocytes due to a reduced turnover rate (Stoykova et al., 1985). In addition, ribosome biogenesis dynamically alters with stages of brain development. For example, the nucleolar number in rat and chicken cerebellar Purkinje neurons increases from the embryonic stage to the post-natal/hatching period (Lafarga et al., 1995). In mice, the synthesis of a few ribosomal proteins (RP), ribosome biogenesis factors, and translation factors are repressed in the neuronal progenitor cells following neural tube closure. The resulting dip in protein synthesis capacity is required for a timed reduction in the rate of proliferation of these cells which, otherwise causes macrocephaly (Chau et al., 2018). However, later during forebrain development, a surge in ribosome biosynthesis promotes dendritic development and arborisation (Slomnicki et al., 2016; Chau et al., 2018). Studies in mouse hippocampal neurons have shown that a moderate depletion of ribosomal proteins S6, S14, or L4, required for subunit export, perturbs dendritic growth and development. This is due to the reduced ribosomal recruitment and translation of BDNF target mRNAs despite having signaling pathways from the TrkB receptors intact (Slomnicki et al., 2016). In addition, the ribosome content is depleted within axons during synaptogenesis (Costa et al., 2019). Together these results highlight that ribosome biogenesis and assembly are regulated in a dynamic and site-specific manner during brain and neuronal development.

Also, neuronal stimulation affects rDNA transcription. For example, 1-h stimulation of auditory nerves results in a significant rise in the rRNA content of chicken cochlear neurons (Hyson and Rubel, 1995). In Aplysia neurons, 5-HT or LTF-inducing stimulus leads to an elevation in pol I-mediated rRNA synthesis. Translocation of the chromatin remodeling protein PARP1, following the activation of the PKA-ERK pathway, mediates this rapid synapse to nucleus signaling (Hernandez et al., 2009; Figure 1). Another study has observed that on NMDA stimulation, AIDA1, a synaptic PSD-interacting protein, translocates from the synapse to the nucleus to regulate nucleolar numbers (Jordan et al., 2007). These observations explain the mechanistic basis of the dynamic communication between the nucleolus and synapse that shape ribosome biogenesis in response to synaptic signaling.

**FIGURE 2:**
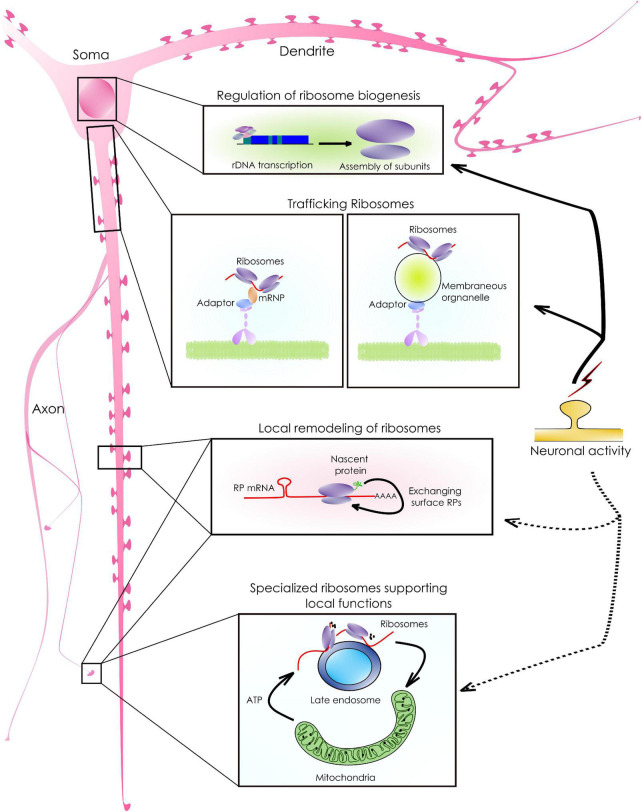
Ribosomal synthesis, sorting, and remodeling to support local functions. As shown in the schematic, neurons have an arborized morphology. mRNA abundant. As a result, the availability of ribosomes and translation machinery in these remote locations is crucial for the abundant local mRNA translation that supports neuronal functions. Following biosynthesis, ribosomal sorting in neurons happens either *via* diffusion or by active transport. The active sorting, is achieved either by associating with mRNPs or by tethering to membrane-bound organelles targeted to distant locations. In addition, existing ribosomes can exchange locally synthesized surface proteins (RPs) giving rise to ribosomes with specialized functions. For example, RP mRNAs with a specific sequence motif are translated into the axons which, can then associate with the surface components of existing ribosomes. Such modifications allow ribosomes to translate a distinct set of transcripts that support local functions.

Following the synthesis of 45s pre-rRNA transcript, several molecules take part in rRNA maturation (Thomson et al., 2013; Sen Gupta et al., 2018). While a key enzyme Nucleolin is involved in transcriptional regulation and cleavage of the 45s rRNA, other critical proteins like Fibrillarin catalyze the modifications of hundreds of bases and backbone residues of the processed rRNA (Boisvert et al., 2007; Sen Gupta et al., 2018). These modifications, such as 2′ *O*-methylations, can influence rRNA secondary structures, the subunit RP compositions, ribosomal association with various RNA-binding proteins (RBPs), and thus allowing ribosomes with specific rRNA modifications to translate a distinct set of mRNA targets (Kondrashov et al., 2011; Polikanov et al., 2015; Simsek et al., 2017; Sloan et al., 2017; D’Souza et al., 2018; Merkurjev et al., 2018; Figure 1). In addition, activity-dependent changes in rRNA modifications can alter global mRNA translation. For example, the induction of experience-dependent plasticity leads to an increased rRNA production and translation in neurons. Neurons express a long nucleolus-specific lncRNA (LONA) that precludes both Nucleolin and Fibrillarin activity, reducing the pro-translational 2′ *O*-methyl marks on rRNA. However, synaptic activation causes degradation of LONA, relieving the inhibition of global translation and upscaling the rRNA production (Li et al., 2018; Figure 1). However, the evidence for other types of rRNA modifications impacting local ribosomal performance in neurons is yet to come. Further experiments can reveal whether individual ribosomes carrying unique combinations of rRNA modifications (an epitranscriptomic code) can influence their functions distinctly (Li and Wang, 2020). Nevertheless, rRNA modifications represent an additional layer of ribosomal regulation in neurons (Figure 2).

## Ribosomal Sorting and Distribution

Localization of ribosomes to subcellular compartments allows neurons to mount a rapid translation response upon stimulation (Figure 2). It is currently believed that while the subunits diffuse freely anywhere in the cell, most assembled ribosomes reach their target location by associating with various RBPs, membrane-less granules, or membrane-bound organelles (Rolls et al., 2002; Fernandopulle et al., 2021). Such association can also lead to the functional compartmentalization of ribosomes. For example, a recent study has reported that the presynaptic ribosomes, tethered to *Rab7a*-containing endosomal vesicles, drive the translation of mRNAs relevant to mitochondrial functions (Cioni et al., 2019; Figure 2). In axonal growth cones, the DCC receptor and its ligand netrin regulate the translation of their target mRNAs by immobilizing the ribosomes locally (Martin, 2010; Tcherkezian et al., 2010; Kim and Martin, 2015; Koppers et al., 2019). These results highlight the importance of spatial confinement of ribosomes for compartmentalizing translation (Reid and Nicchitta, 2015; Cioni et al., 2019).

A large fraction of the cytosolic ribosome functions as polyribosomes. They often reach their target locations by piggybacking partner mRNPs or transporting granules (Fernandopulle et al., 2021). For example, TDP43 and FMRP/FUS containing transport granules are shown to carry polyribosomes to neuronal axons and dendrites, respectively (Miyashiro et al., 2003; Simsek et al., 2017; Thelen and Kye, 2020; Nagano et al., 2020). Both motor proteins such as Kinesin I, II, Myosin II, and V and adaptor proteins like RACK1 help in the precise targeting of polysomes (Hirokawa et al., 2010; Ceci et al., 2012; Spillane et al., 2013). However, given the heterogeneous properties of ribosome-associated mRNPs and the contradictory nature of evidence, it is not clear whether moving polysomes can translate actively (Wu et al., 2015; Katz et al., 2016; Mateju et al., 2020). This necessitates the study of the translatability of moving polysomes in greater depth.

While ample evidence supports the polyribosomal translation of mRNAs at somatic and dendritic compartments, evidence for axonal ribosomes has been hard to come by for a long period (Ostroff et al., 2002, 2018, 2017; Biever et al., 2019). Eventually, improved paradigms have allowed the detection of mRNAs, rRNAs, actively translating polysomes, ER-Golgi-mediated protein synthesis, and their membrane targeting in axons (Giuditta et al., 1968, 1980, 1986; Tennyson, 1970; Bassell et al., 1998; Willis, 2005; Merianda et al., 2009). Recent investigations have discovered additional mechanisms that aid in axonal ribosomal homeostasis. For example, studies on injured sciatic nerves have shown that polyribosomes are transferred to the axons from the neighboring Schwann cells through tunneling nanotubes or exosomes (Court et al., 2008). In addition, a few other current studies suggest that in the axons, monosome-mediated translation is abundant compared to polysomes (Biever et al., 2019, 2020; Koltun et al., 2020). These findings imply that neurons utilize a range of mechanisms to supply ribosomes to axons, where the translation program is modified uniquely to meet the local protein demands. The fact that several stimulations uniquely impact axonal translation corroborates this idea further (Hafner et al., 2019; Koltun et al., 2020).

## Local Remodeling of Ribosomes

Ribosomal subunits are composed of ∼80 ribosomal proteins and 4 different rRNA species. Conventionally it is believed that the protein or rRNA composition of the ribosomal population within a cell is consistent over time (Ferretti and Karbstein, 2019). However, multiple recent reports suggest otherwise. Studies in various non-neuronal systems have detected differential transcription and splicing of RP mRNAs and change in the stoichiometry of ribosomal core proteins across a range of physiological states (Bortoluzzi et al., 2001; Sharov et al., 2003; Richards, 2004; Adjaye et al., 2005; Kondrashov et al., 2011; Slavov et al., 2015). Some of these studies also show that ribosomes with distinct RP compositions can preferentially translate a subpool of mRNAs. For example, ribosomes containing RPL10A/uL1 protein selectively translate the internal ribosome entry site (IRES)-containing mRNAs (Shi et al., 2017). Interestingly, neurons also contain a large proportion of RPL10A containing ribosomes at the dendrites (Koltun et al., 2020; Sun et al., 2021). However, it is not known whether they translate a selected set of mRNAs in these compartments. Other than the RP composition, their posttranslational modifications are necessary for ribosomal function and can be a source of significant functional variability (Belin et al., 2010; Loenarz et al., 2014; Simsek and Barna, 2017). For example, in RPL12/uS23, hydroxylation of a proline residue is required for polysome formation, without which, human patients develop microcephaly and hearing loss (Loenarz et al., 2014). In addition, phosphorylated forms of various ribosomal proteins are studied widely in the context of synaptic signaling and are implicated in Parkinson’s disease (Simsek and Barna, 2017; Martin et al., 2011). Besides, a combination of methylated, acetylated, and ubiquitylated ribosomal proteins can define some form of post-translational modifications code (PTM code) that can direct a ribosome to function uniquely (Nesterchuk et al., 2011; Simsek and Barna, 2017). Such functional specifications corroborate the “ribosome filter hypothesis” that considers ribosomes as active components of the gene regulatory framework (Mauro and Edelman, 2002).

In neurons, a consistent yet intriguing finding has been an abundance of RP mRNAs in the neuronal branches away from the cell body (Poon et al., 2006; Cajigas et al., 2012; Rangaraju et al., 2017). The targeting of these mRNAs to the distal processes cannot be explained by the Brownian diffusion and requires a reassessment of their physiological roles in these compartments. Also, there is evidence to support the ubiquitous synthesis of ribosomal proteins at distant locations, where they can physically associate with the pre-existing ribosomes. For example, RP mRNAs with CUIC-sequence motifs are translated locally and are incorporated into the axonal ribosomes required to translate critical mRNAs for axonal maintenance and branching (Shigeoka et al., 2019). The translation of RP mRNAs in axons requires the survival of the motor neuron (SMN) protein in the absence of which, axonal ribosomal content dips by 27% (Fallini et al., 2012, 2016). In dendrites, 17 ribosomal proteins are synthesized locally while 12 of them are incorporated into the existing ribosomes rapidly (Fusco et al., 2021). These proteins are short-lived and are associated with the solvent-accessible surfaces of ribosomes (Shigeoka et al., 2019; Fusco et al., 2021). Such dynamic exchanges of subunit proteins are necessary for the maintenance, repair, or modification of their functions in the local compartments (Figure 2). Interestingly, ribosomes are often located at both dendritic and axonal branch points. Here, they colocalize with mitochondria and other RBPs to translate mRNAs critical for stabilizing branches (Cui-Wang et al., 2012; Spillane et al., 2013).

Despite our knowledge of dendritic and axonal translation, we know relatively little about the synthesis of ribosomal proteins at the synapse. Ribosome profiling from cortical synaptoneurosomes has revealed that a few RP mRNAs are synthesized locally at the synapse upon NMDAR stimulation (Kuzniewska et al., 2020). Whether they can modulate existing ribosomal function requires verification. Moreover, since solitary monosomes translate the bulk of the synaptic mRNAs, especially with shorter ORFs (Biever et al., 2020; Koltun et al., 2020), conceptually it is feasible to selectively alter the translation of such mRNAs by modifying single ribosomes. Whether such mechanisms operate at the synapse requires validation through experimentation.

Also, these studies have opened up a flurry of other questions that remain unanswered. For instance, given the distinct translation states of various neuronal compartments, whether ribosomes with unique RP compositions or rRNA modifications populate these compartments differentially remains to be seen. In addition, it is not clear whether the type of synaptic activity (i.e., excitatory vs. inhibitory) has distinct impacts on the ribosomal properties. Besides, within a synapse, there can be multiple functional zones with heterogeneous molecular compositions (Nanguneri et al., 2019; Venkatesan et al., 2020). Whether ribosomes proximal to these zones function uniquely is not understood. Also, specific biochemical mechanisms that control ribosome-RBP association needs to be identified. Intriguingly, a large part of the variability in protein expression is contributed by the stochasticity of translation elongation (Datta and Seed, 2018; Koltun et al., 2020; Sun et al., 2021). Whether rRNA modifications and subunit protein compositions contribute to the stochastic aspects of protein synthesis are not well characterized. Also, it needs to be investigated whether specialized ribosomes necessarily need to function as monosomes. In the case of a polysome, do all ribosomes harbor similar subunit compositions? Whether sequence and structural features of mRNAs dictate their affinity toward ribosomes? How does the presence or absence of an RBP affect ribosome-mRNA recognition? Some of these questions need to be addressed thoroughly for a better understanding of translation regulation in neurons. Besides, mRNA nucleotides often undergo chemical modifications posttranscriptionally, known as the epitranscriptomic changes, which represent an additional layer of translation regulation. A large number of synaptic mRNAs harbor critical modifications such as N^6^-methyladenosine, m^5^-cytosine, etc., because of the actions of a group of depositing (writer), binding (reader), and removing (eraser) enzymes (Flamand and Meyer, 2019). For example, almost 3,000 synaptic mRNAs are m^6^A methylated (Merkurjev et al., 2018). These modifications either facilitate or occlude the binding of modified mRNAs to RBPs and translation machinery, thus, tuning translation kinetics (Merkurjev et al., 2018; Li et al., 2019). A study has shown in the past that mRNA modification such as N^6^-methyladenosine (m^6^A) alters the recognition time and binding probability of a cognate tRNA to an mRNA codon and consequently alters elongation dynamics (Choi et al., 2016). Also, it is established that the absence of these RNA modifying enzymes affects synaptic translation and plasticity (Li et al., 2019). Further insight would clarify whether the deficiency of such enzymes impacts the ribosome-mRNA recognition, binding, and translatability of these mRNAs. Finally, how these processes are altered in pathological conditions or with aging should be monitored. Nonetheless, on-site remodeling of ribosomes through various mechanisms represents an exciting ramification of our understanding of spatiotemporal gene expression regulation in neurons.

## Local Factors That Affect Translation Kinetics

For the holistic understanding of neuronal translation, numerous other factors need considerations. Since distant branches with smaller diameters present significant diffusion barriers, local factors are bound to influence ribosomal function. However, such studies are limited in the neuronal context. We discuss a few components from the local microenvironment that could be critical in our opinion to shape ribosomal function.

### Concentration of Cations

#### K^+^

K^+^ is a major intracellular monovalent cation in neurons. Recent studies have revealed the role of K^+^ ions as integral components of the ribosomal structure (Khatter et al., 2015; Rozov et al., 2019). In eukaryotic ribosomes, K^+^ ions strengthen the intersubunit interactions and mediate the interactions between tRNAs and ribosomal components (Wilson and Cate, 2012; Khatter et al., 2015). This is unlike the prokaryotic ribosomes, where the role of Mg^2+^ ions is more prominent (Ramakrishnan, 2002; Nierhaus, 2014). Intriguingly, recent studies from prokaryotic ribosomes also have suggested diverse roles for K^+^ ions (Rozov et al., 2019). However, our current understanding of the influence of K^+^ ions upon ribosomal performance in the cellular context is limited. In the case of hippocampal neurons, while the steady-state concentration of K^+^ [(K^+^)_*i*_] is ∼140 mM, it can reduce rapidly up to almost ∼43 percent following glutamate-mediated depolarization (Ballanyi et al., 1984; Müller and Somjen, 2000; Somjen and Müller, 2000; Nierhaus, 2014; Shen et al., 2019). Similar observations were made for carbachol or GABA treatment [∼20% and 8.5% (K^+^)_*i*_ respectively (Ballanyi et al., 1984)]. Such changes would be pronounced in the small volume compartments like dendritic spines or axonal growth cones and are likely to impact ribosomal subunit interactions and subsequent functions. Considering a large body of evidence pointing to the fact that neuronal depolarization can affect protein synthesis (Sutton et al., 2004; Hsu et al., 2015; Kos et al., 2016; Brigidi et al., 2019; Dastidar et al., 2020), it is worth investigating whether such effects are mediated by the changes in [K^+^]_*i*_. In addition, multiple reports suggest altered neuronal firing and membrane properties upon treatment with global protein synthesis inhibitors (Kleim et al., 2003; Sharma et al., 2012; Scavuzzo et al., 2019). Whether this effect is due to the release of a large amount of ribosome-bound K^+^ ions following the inhibitor actions needs verification.

#### Mg^2+^

Magnesium is an abundant divalent cation within mammalian cells. In neurons, the intracellular Mg^2+^ concentration ranges between 17–20 mM (Romani, 2011). Interestingly, the concentration difference of Mg^2+^ ions across the plasma membrane is only twofold (compared to 20,000-fold of calcium). There are various mechanisms by which Mg^2+^ ions are stored in a cell, of which ribosome-bound Mg^2+^ pool represents a considerable fraction. In humans, each ribosome is bound to 239 Mg^2+^ ions (Khatter et al., 2015). Studies of diverse backgrounds have revealed that Mg^2+^ is a small electron-dense ion that helps in stabilizing the negative charges of the rRNA backbone residues (counterions) (Nierhaus, 2014). Given a neuron can have as many as 10^6^–10^7^ ribosomes, the amount of ribosome-bound Mg^2+^ is substantial. However, due to the shallow concentration gradient of Mg^2+^ across the membrane and the continuous exchange between their bound and the free form in the cytosol, changes in extracellular Mg^2+^ availability can severely impact the intracellular Mg^2+^ concentration and ribosomal functions. Also, the Mg^2+^ deficiency within neurons can be sensed by mTOR signaling that can alter translation in several ways (Shindo et al., 2020).

#### Ca^2+^ and Mn^2+^

Apart from being a cation, Ca^2+^ acts as an important intracellular second messenger in neurons. However, its role in the context of neuronal ribosomes is less studied. Earlier observations on purified E. coli ribosomes have established that the presence of Ca^2+^ in the reaction buffer improves ribosomal performance (Gordon and Lipmann, 1967). However, later studies have found that another divalent cation Mn^2+^ can substitute Mg^2+^ in both subunits while Ca^2+^ could do so only in the small subunit (Weiss et al., 1973). In sync with these observations, Mg^2+^ depletion in pituitary GH3 cells leads to a complete abolishment of polysomes. However, replenishing Ca^2+^ quickly recovers the polysomal functions, highlighting a positive influence of calcium over eukaryotic polysomes (Chin et al., 1987). In addition, Ca^2+^ promotes the association between purified human ribosomes and the Ca^2+^-sensitive protein Calmodulin (Behnen et al., 2012). Interestingly, at the neuronal synapse, Ca^2+^ activates several EF-hand-containing proteins such as Calmodulin, Caldendrin, Calbindin, Calreticulin, Calneuron, and others (Chard et al., 1995; Seidenbecher et al., 1998; Pangršič et al., 2015; Mundhenk et al., 2019). Some of them, such as Calneuron 1 and 2, have the highest affinity toward Ca^2+^. Functionally, they are implicated in Golgi to plasma membrane protein trafficking (Mundhenk et al., 2019). However, not much is known whether they can influence protein synthesis. Since neuronal activation alters intracellular Ca^2+^ levels, an exciting direction would be to investigate how these various Ca^2+^-binding proteins act together to regulate ribosomal functions following neuronal stimulation.

### ATP Level

Intracellular ATP concentration is a major rate-limiting factor for almost all anabolic pathways. ATP level is particularly important for protein synthesis due to multiple ATP-consuming steps in mRNA translation (Schwanhäusser et al., 2011). Our previous study has demonstrated that neuronal activity-induced protein synthesis is responsible for a significant ATP expenditure (Dastidar et al., 2020). Others have observed that a deficiency in energy biosynthesis can attenuate activity-induced synaptic translation (Rangaraju et al., 2019). Moreover, perturbing mitochondrial function that colocalizes with the translational hotspots at the neurite branch points impairs brach-point protein synthesis and branch stabilization (Hill et al., 2012; Spillane et al., 2013; Li et al., 2004). Conversely, new proteins can support mitochondrial function locally. For example, local translation of lamin B2 at the retinal ganglionic axons of *Xenopus* maintains mitochondrial morphology and function (Yoon et al., 2012). In this context, an intriguing observation has been that an excess of intracellular ATP can negatively impact translation by binding additional Mg^2+^ ions, thus limiting Mg^2+^-dependent ribosomal assembly (Pontes et al., 2015). Therefore, a coordinated regulation between protein synthesis and energy biosynthesis is necessary for neuronal functions and can be achieved by the actions of intracellular metabolic sensors like phosphofructokinase 1 (PFK1) or AMP-activated protein kinase (AMPK) (Jang et al., 2016; Marinangeli et al., 2018; Dastidar et al., 2020). In general, the interplay between metabolic and translation regulatory pathways remains incompletely understood and would be an active area of future research.

### tRNA Availability

Transfer RNAs (tRNA) canonically function as adapter molecules during mRNA translation. The availability of tRNAs, therefore, is one of the rate-limiting parameters of the ribosomal function. The redundancy in the genetic code allows most amino acids to be encoded by multiple codons. Also, these codons are distributed non-randomly along the length of an mRNA message (Komar, 2016) and are related to the decoding times for each codon (Gardin et al., 2014; Quax et al., 2015). Indeed eukaryotic ribosomes can accommodate frequent codons more rapidly at the A site than the rare codons (Gardin et al., 2014). The codon usage bias has been estimated to account for 30% of the variation in mRNA-protein correlation in human cells (Schwanhäusser et al., 2011). Also, the difference in decoding time determines the speed of polypeptide emergence and co-translational protein folding (Waudby et al., 2019). In other words, any change in the mRNA codons influences their average decoding time, the rate of ribosomal translocation, the rate of polypeptide emergence, and hence the folding probability of a protein toward its native state. Since tRNA availability influences the average decoding time, the rapid activity-induced translation response would require various tRNA species to be readily available within local compartments. Toward this end, the presence of tRNAs was detected within neuronal dendrites back in 1996 (Tiedge and Brosius, 1996). In addition, a more recent study has determined their kinetics inside neurons (Koltun et al., 2020). In this study, the exogenously labeled tRNAs introduced in the cortical neurons showed punctate structures. In general, the tRNA puncta were bidirectionally transported in the dendrites and a fraction of them (generally larger in size) could be destabilized with puromycin, a translation inhibitor that disengages elongating ribosomes, indicating they were part of actively translating complexes. However, chemical LTP induction led to a further increase in these large tRNA aggregation and potentially mRNA translation (Koltun et al., 2020).

Despite the advances, appreciating various dimensions of tRNA function in neuronal mRNA translation would require more information. In particular, how the concentration of individual tRNA species affects ribosomal processivity is not established clearly. Also, considering the differences in decoding time for various codons (Gardin et al., 2014), it would be compelling to probe whether mRNAs in a compartment show distinct codon usage patterns and whether the tRNAs for those codons are enriched in those locations. Finally, given the rising evidence of on-site ribosomal remodeling, one would be intrigued to know if the remodeled ribosomes prefer to bind specific tRNA species and translate mRNAs with distinct codon-usage patterns.

### *In cellulo* Dynamics of Neuronal Ribosomes

Decades of work in biochemistry and insight from high-resolution structures have elucidated the founding principles of mRNA translation and ribosomal function. But, these approaches provide very little dynamic information in the cellular realm. More recent studies based on single-molecule tracking have been instrumental in describing the dynamic properties of ribosomes and mRNAs within cells (Volkov and Johansson, 2019). Both prokaryotic and eukaryotic ribosomes have been visualized by tagging RPs with fluorescent proteins (Katz et al., 2016; Bayas et al., 2018). These experiments have been critical in understanding the population behavior of ribosomes arising from thousands of single-molecule detection events. Especially, live tracking of ribosomal subunits allows correlating their dynamics to mRNA translation within a cell (Prabhakar et al., 2019; Volkov and Johansson, 2019). For example, single-molecule diffusion studies on E. coli ribosomes have found that while the subunits themselves can diffuse freely across the entire cell, elongating ribosomes are excluded from the nucleoid (Sanamrad et al., 2014). Besides, simultaneous tracking of mRNPs and ribosomal particles on mouse fibroblast lines has revealed that polysome-associated mRNPs move slower than the mRNPs alone. Ribosomes in these cells show two prominent diffusional states (Katz et al., 2016). While the majority display relatively less mobility, a smaller freely diffusing fraction moves more rapidly (Katz et al., 2016; Donlin-Asp et al., 2021). Interestingly, treatment with puromycin increases the mobility of both ribosomes and mRNPs in these cells. Another study noted a similar effect of puromycin treatment on the mRNP dynamics in neurons (Donlin-Asp et al., 2021). These results suggest that polyribosomes impede the movement of mRNP complexes by making them heavier while destabilizing them with puromycin increases their mobility (Katz et al., 2016). However, one problem with single-molecule experiments is that the fluorescent proteins coexpressed with the proteins of interest often show moderate brightness and photostability (Chudakov et al., 2010; Volkov and Johansson, 2019). In addition, the presence of a large number of subdiffraction ribosomal particles can complicate the detection and analysis of single-molecule trajectories (Volkov and Johansson, 2019). To this end, a combination of photoactivable or photoswitchable fluorescent proteins/fluorophores and imaging techniques that can overcome the diffraction barrier such as STED, RESOLFT, and PALM/STORM have enabled molecular tracking with relatively lesser complications (Manley et al., 2008; Nair et al., 2013; Shcherbakova et al., 2014; Kedia et al., 2021). For example, observations made through sptPALM trajectories in migrating mouse fibroblast cells describe that ribosomes near focal adhesions of the leading edge show much-confined movement compared to elsewhere in the cytosol. Since ribosomes tend to dwell more at the site of translation, such interchanges between diffusion states are envisioned to drive compartmentalization of protein synthesis (Katz et al., 2016).

**FIGURE 3:**
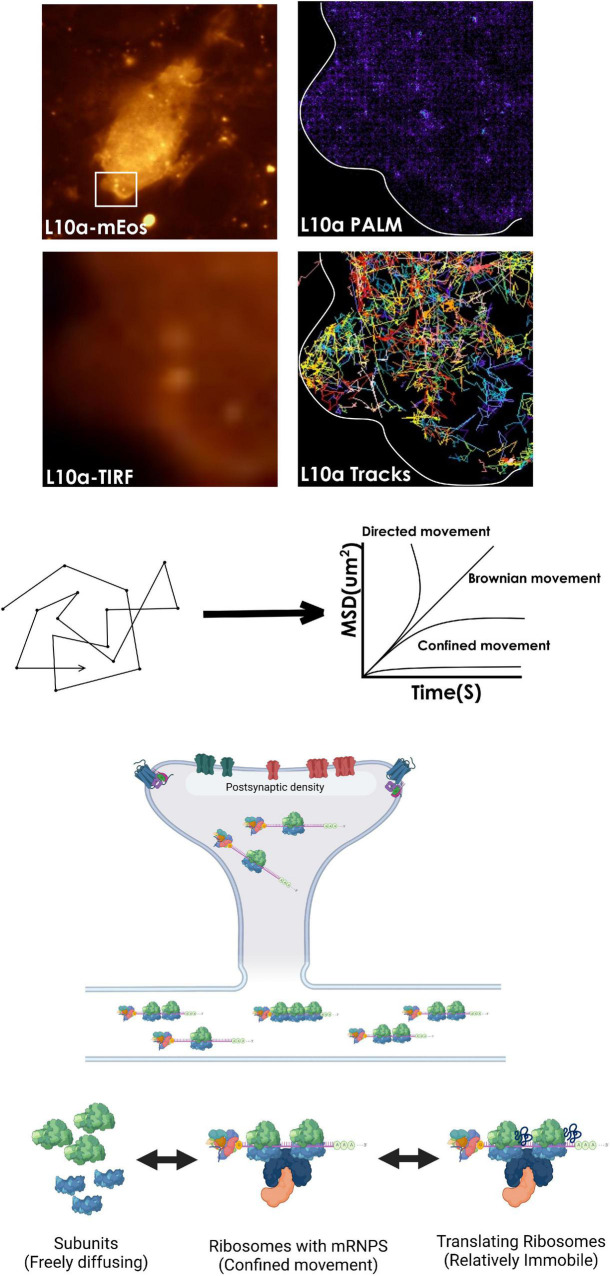
Tracking ribosomal mobility for locating “hotspots” of active translation. Visualizing and tracking ribosomal subunits at the single-particle level is possible by tagging a subunit protein such as L10a with photoactivable fluorescent proteins such as mEos within a cell. Analysis of their mean square displacement (MSD) trajectories of single protein molecules can then be used to calculate the diffusion coefficients of various ribosomal populations, that switch between diffusional states while functioning within cells. In addition, perturbations with pharmacological agents that destabilize ribosomes can confirm their engagement in active translation. Also, an HMM-based analysis of the trajectories can be used to reveal the mechanisms underpinning the conversion between diffusional states of ribosomes (not shown).

Despite considerable progress, much of our understanding of the dynamics of individual ribosomes in neurons remain elusive. Considering the large variability in the mRNP composition, kinetics, and biochemical properties (Formicola et al., 2019; Tauber et al., 2020), it is necessary to probe how mRNP properties affect ribosomal movement and function. In the same context, it would be useful to generate a neuron-wide ribosomal mobility map with nanometer precision. In addition, compartments like dendritic spines or growth cones show non-homogeneous molecular distribution and organization (Frost et al., 2010; Igarashi, 2019). While functional zones like post-synaptic density (PSD) are protein-dense regions, other areas of a spine are more dynamic with varied molecular compositions (Kaizuka and Takumi, 2018; Venkatesan et al., 2020; Helm et al., 2021). Interestingly, similar to focal adhesions, PSD is also reported to associate with polyribosomes, RBPs and act as a platform of translation (Ostroff et al., 2017; Yoon et al., 2016; Zhang et al., 2012). However, ribosomal diffusion kinetics in and around PSD has not been examined yet. Using single-molecule localization microscopy (SMLM), it is now possible to track the mean square displacement of ribosomes and calculate their diffusion parameters from HMM-bayesian modeling. Given the strong correlation between ribosomal confinement and active translation, the analysis can reveal the spatial distribution of translation “hotspots” within spines in live neurons (Monnier et al., 2015; Katz et al., 2016; Volkov and Johansson, 2019; Figure 3). In addition, we are yet to know how neuronal stimulation may impact ribosomal localization and their dynamics within spines (Ostroff et al., 2017). As discussed before, synaptic molecules often organize into functional zones by forming nanoclusters. Signaling through these nanodomains is crucial for synaptic plasticity and protein synthesis (Nair et al., 2013; Goncalves et al., 2020; Sun et al., 2021). For example, GluA1 or GluA2 and PSD 95 nanodomains at the dendritic spines are the hubs of intracellular signaling. The efficacy of signaling depends on nanodomain sizes and the localization of receptors in these clusters (Nair et al., 2013; Tang et al., 2016; Nanguneri et al., 2019; Venkatesan et al., 2020). In addition, it is observed that within translationally active spines, PSD microdomains often associate with newly synthesized proteins which, in turn, are closely apposed to ribosomes (Sun et al., 2021). However, little is known whether the molecular organization of the PSD can sequester or trap ribosomes to initiate localized translation. Also, the precise nanometer level localization maps of receptors, PSD, ribosome distribution, and protein synthesis hotspots together are not available yet. These finer insights into spatio-temporal correlation and regulation of ribosomal functions would be valuable to understand the stochastic constraints that control local rates of translation. In parallel, direct visualization of single ribosomes and their kinetics can verify whether their sequestration at the PSD is necessary for localized translation. Perturbing nanodomain properties using pharmacochemical or optogenetic approaches followed by ribo-tracking can indeed delineate the relationship between the molecular organization, and protein synthesis at the individual spine level. Altogether, these approaches provide us with the opportunity to answer the previously underexplored questions of mRNA translation. They also allow us to appreciate the tremendous variability in ribosomal functions within neurons with unprecedented details.

## Concluding Remarks

Proteome remodeling is a critical component of the neuronal response to several incoming stimuli. The complex morphology of neurons requires the remodeling to be done locally in a compartmentalized manner. Together with various other mechanisms, ribosomal modulation (through biogenesis, sorting, local remodeling, and dynamic properties) provides a way to create variability in protein expression. However, the spatiotemporal kinetics of such changes and their relation with synaptic signaling remains to be determined. During memory formation, information is stored from shorter to longer time scales at the synapse. Yet the mechanistic connections between the various phases of memory formation remain to be uncovered. Considering signaling events and instantaneous molecular organizations encode information for a short period while protein synthesis dictates its long-term storage, a rigorous connection between the events of these two timescales can explain how memories are stored permanently from their shorter labile versions. With the emergence of suitable technologies with high spatial and temporal precisions, we are finally in a position to address some of these pressing questions.

## Author Contributions

SD conceptualized and designed the manuscript. Both authors wrote and edited the manuscript and approved the submitted version.

## Conflict of Interest

The authors declare that the research was conducted in the absence of any commercial or financial relationships that could be construed as a potential conflict of interest.

## Publisher’s Note

All claims expressed in this article are solely those of the authors and do not necessarily represent those of their affiliated organizations, or those of the publisher, the editors and the reviewers. Any product that may be evaluated in this article, or claim that may be made by its manufacturer, is not guaranteed or endorsed by the publisher.
